# Working Hypothesis for Glucose Metabolism and SARS-CoV-2 Replication: Interplay Between the Hexosamine Pathway and Interferon RF5 Triggering Hyperinflammation. Role of BCG Vaccine?

**DOI:** 10.3389/fendo.2020.00514

**Published:** 2020-07-07

**Authors:** Hugo A. Laviada-Molina, Irene Leal-Berumen, Ernesto Rodriguez-Ayala, Raul A. Bastarrachea

**Affiliations:** ^1^Escuela de Ciencias de la Salud, Universidad Marista de Mérida, Mérida, México; ^2^Facultad de Medicina y Ciencias Biomédicas, Universidad Autónoma de Chihuahua, Chihuahua, México; ^3^Centro de Investigación en Ciencias de la Salud (CICSA), Facultad de Ciencias de la Salud, Universidad Anáhuac Norte, Naucalpan de Juárez, México; ^4^Population Health Program, Texas Biomedical Research Institute and Southwest National Primate Research Center (SNPRC), San Antonio, TX, United States

**Keywords:** HB pathway, IRF5, SARS-CoV-2, BCG, cytokine storm

## Introduction

**Figure 1:**
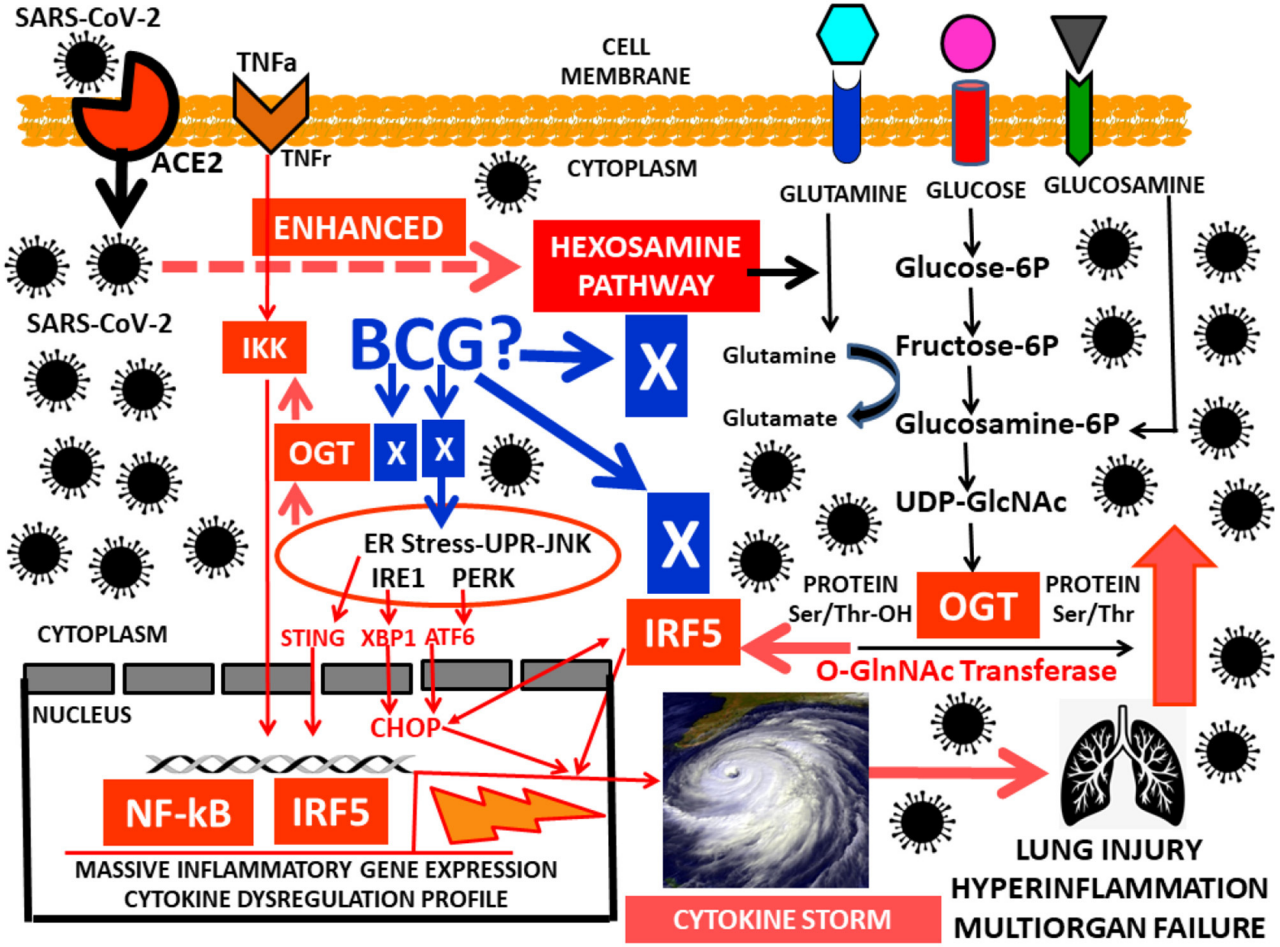
Work in progress hypothesis for the hyperinflammatory HBP-GlcNAc-OGT–IRF5-interferon-SARS-CoV-2 pathway leading to the cytokine storm.

Respiratory epithelial cells, dendritic cells (DCs) and macrophages (1) secrete low levels of the antiviral factor interferons (IFNs) (2) and high levels of proinflammatory cytokines such as tumor necrosis factor alpha (TNFα), IL-6, and IL-1β, IP-10, MCP-3, characterizing the pathophysiologic features of severe acute respiratory syndrome (ARDS) induced by SARS-CoV-2 infection (3). Researchers hypothesized that after COVID-19 infects human cells, the virus utilizes an excess of glucose for a fast viral replication from the hexosamine biosynthetic pathway (HBP) hijacking substrates from the metabolic environment. This process induces overexpression of interferon IRF5, leading to a massive inflammatory gene overexpression, endoplasmic reticulum (ER) stress, and cytokine dysregulation profile. This deleterious cytokine overproduction is referred to as the cytokine storm (Figure 1). It leads to an increased risk of vascular hyperpermeability, multiorgan failure, and hyperinflammation (4, 5). Little is known about the molecular immunometabolic mechanism that triggers the uncontrolled surge in cytokine secretion and the cellular regulation of the hyperinflammatory cascade intertwined with the COVID-19 viral replication.

## Chronic Low-Grade Subclinical Inflammation: Underlying Mechanism Enhacing the COVID-19 Cytokine Storm in the Presence of Dysglycemia

Older people, immunocompromised, diabetics and/or hypertensives are more likely to develop the cytokine storm. Dysglycemia is characterized by glucose intolerance and insulin resistance (6, 7). Additionally, aberrant expression of proinflammatory cytokines adds to the toxic milieu of dysglycemia (8). These immunometabolic disturbances also include hyperglycemic posprandial peaks perhaps exacerbating the excess of proinflammatory cytokines in COVID-19 (9). However, a subgroup of symptom-free non-diabetic, overweight individuals with mild body fat accumulation and insulin resistance (IR) and/or prediabetes (10), and a second subgroup of symptom-free normal weight metabolically unhealthy subjects have a 3-fold higher risk of all-cause mortality and/or cardiovascular events (11). These two subgroups are also at high-risk to develop the cytokine storm. They have in common a chronic systemic low-grade subclinical proinflammatory (CLGSPI) state (12) characterized by adipose tissue dysfunction (ATdys) (13) and macrophage polarization in adipose tissue, low adiponectin levels, cytokines and circulating inflammatory C-reactive protein (CRP) that perpetuates this deleterious CLGSPI and promotes insulin resistance (14). These subgroups of individuals with underlying but undetected postprandial dysglycemia, CLGSPI and ATdys present a proinflamatory pathology that is highly underestimated and rarely diagnosed among symptom-free individuals in the practice setting. This undetected deleterious chronic low-grade proinflamatory scenario perhaps together with postprandial glucose excursions is proposed here as the underlying mechanism enhancing the hyperinflammatory state in these infected symptom-free subjects with COVID-19 triggering multi-organ failure.

## Glucose Metabolism, the Hexosamine Pathway, IRF5, SARS-CoV-2 Replication and Cytokine Storm

After glucose uptake, in addition to glycolysis, glucose metabolism generates UDP-GlcNAc (uridinediphosphate- β-D-N- acetylglucosamine) via the HBP pathway. The HBP links cellular signaling and gene expression to glucose, amino acid, fatty acid and nucleotide metabolism (15, 16). It is regulated by the enzymes OGT (O-GlcNAc transferase) and OGA (O-GlcNAcase), that catalyze the addition and removal of GlcNAc on proteins (17). UDP-GlcNAc is a substrate for N-glycosylation, a process important for protein folding within the ER (18, 19) (Figure 1). The HBP- O-GlcNAc pathway has also been characterized as a major contributor to the deleterious effects of dysglycemic states, also influencing cellular proliferation. These dysglycemic states, mainly overt hyperglycemia, have a significant contribution in oncogenesis, tumor progression and fatal outcomes, indicating that there is a link between glucose metabolic disorders and tumor growth of cancer cells (20, 21). Similar biological characteristics are found in viruses. They rearrange the metabolic environment in the infected cells to facilitate virus replication. Indeed, virus-infected cells increase glycolytic metabolism to secure precursors for an increased biosynthesis (lipids, nucleotides) to optimize virus production and replication (22, 23).

Nuclear factor-kB (NF-kB) and IKK are associated with metabolic disorders (24), also playing important roles in inflammatory and immune responses (25). O-GlcNAcylation regulates direct modification of transcription factors such as NF-kB (26, 27). Cells use the HBP and OGT to potentiate gene expression through NF-kB as a glucose-responsive transcription factor in response to TNFα (28) (Figure 1). The interferon-regulatory factor (IRF) family plays a critical role in regulating the immune system, the innate immune response and the development of immune cells. IRFs are primarily implicated in antiviral responses and interferon production. IRFs also have key functions in the regulation of metabolism (29). Among the IRF family members, Interferon Regulatory Factor 5 (IRF5) is a key player in inflammation (30). IRF5 mediates induction of proinflammatory cytokines such as interleukin-6 (IL-6), IL-12, IL-23 and TNFα, is involved in the recruitment of inflammatory genes with NF-kB, and in determining the inflammatory macrophage phenotype (31). Host protection by IRF5 is achieved through its role in the nucleus triggering transcriptional activation of proinflammatory type I interferon (IFN), promotion of apoptosis-related genes and regulation of cytokines involved in cell survival, growth, proliferation, and differentiation (32). ER stress can result in the accumulation of unfolded proteins, triggering the unfolded protein response (UPR), an adaptive reaction that reduces unfolded protein load to maintain cell viability and function, capable of sensing dangerous aggressions and reverse them by influencing the immune response through interferon production (33).

Cells infected with viruses induce ER stress and stimulate strong interferon responses (34). Viral replication is inhibited by an interferon-regulated gene product, the double-stranded RNA-dependent protein kinase (PKR). Interferon employs its antiviral properties by activating PKR, thereby inhibiting viral replication. ER stress is a critical component in the response against viral infections. A prolonged ER stress triggers apoptosis. Therefore, the task of a virus is to overcome the interferon response involving PKR and manipulate the unfolded protein response (UPR) to facilitate viral replication and cause disease (34). A recent study demonstrated that influenza A virus (IAV) was able to induce a cytokine storm via interferon (IFN) regulatory factor−5 (IRF5) through glucose metabolism utilization and an increase in O-GlcNAc signaling, demonstrating that the HBP– *O*-GlcNAc signaling pathway in influenza A virus (IAV) promoted a massive inflammatory cytokine overexpression (35). SARS-CoV-2 infects human cells also leading perhaps to an excess of glucose utilization (16). Indeed, the HBP pathway activated in IAV infections generates UDP-N-Acetylglucosamine (UDP-GlcNAc), substrate for the key enzyme for protein O-GlcNAcylation O-GlcNAc transferase (OGT) (15). This enzyme has a strong binding affinity to signaling protein interferon regulatory factor 5 (IRF5) (35). Viral infections such as IAV and SARS-CoV-2 may create an excess in IRF5, leading to ER stress and rapid ubiquitination, triggering an excess of cytokine overproduction, hyperinflammation and multiorgan failure (36) (Figure 1). Another study identified a molecular mechanism by which HBP-mediated O-GlcNAcylation regulates mitochondrial antiviral signaling protein (MAVS) function (37) and highlighted the importance of glucose metabolism in antiviral innate immunity (38).

## Could BCG Avoid the Hijacking of the HBP-O-GlcNAc-OGT Complex by SARS-CoV-2 to Restore Immunity and Balance Cellular Metabolism?

BCG-treated type 1 diabetes (T1D) individuals showed a durable lowering of HbA1c and glucose (39). BCG appears to switch the immune system of T1Ds from high oxidative phosphorylation to augmented glycolysis, a systemic metabolic shift that allows cells to consume large amounts of glucose to safely lower hyperglycemia (39, 40). The authors were able to reset the immune system to a state of increased glycolysis at the cellular level through turning on T regulatory (Treg) cells. The BCG effect on immune metabolism apparently accelerated glucose utilization through increased glycolysis, a high-glucose-transport process through the pentose phosphate shunt, instead of using the Krebs cycle for oxidative phosphorylation (40). The immune effects of BCG in T1D relates to the autoimmune environment comprising too few suppressive T regulatory (Treg) cells and too many cytotoxic T lymphocytes (CTLs). With BCG treatment, Treg cell expansion and augmented function occurred, and CTLs died thus restoring the immune balance toward normal at the autoimmune site (41). Figure 1 shows multiple potential molecular targets for BCG activity in COVID-19 infected individuals.

Based on the immunomodulatory roles of mycobacteria, an effect from BCG vaccination on the spread and severity of COVID-19 dissemination in different countries could have occurred (42). Researchers classified countries into 2 groups according to presence or absence of BCG vaccination in their routine vaccine schedules and obtained confirmed numbers of COVID-19 cases and deaths from the World Health Organization (WHO). They found that the mean of cases per population ratio was statistically significantly lower in BCG vaccinated countries when compared to BCG-non-vaccinated countries, also finding that both the mean deaths per population and the deaths per cases ratio were significantly lower in BCG-vaccinated countries. They concluded that the cessation of BCG vaccination in several countries within the last few decades should be reanalyzed given its impact regarding the immune response to hypothetical viral pandemics we might face in the future on BCG-non-vaccinated young individuals (42–44). Vaccination with BCG triggers a memory-like response in innate immune cells known as “trained immunity” associated to an epigenetic reprogramming mechanism in both humans and mice (45). However, some authors advice caution when interpreting data on Covid-19 incidence and BCG vaccination. Further research is definitively needed to prove that the BCG vaccine confers protection against COVID-19. Indeed, the current state of knowledge does not provide sufficient evidence (46). A trial for BCG vaccination to reduce the impact of COVID-19 in Australian healthcare workers (BRACE) has been set to investigate whether BCG vaccination protects against COVID-19 (mcri.edu.au/BRACE).

## Discussion

As depicted in Figure 1, we speculate as a work in progress hypothesis that after SARS-CoV-2 have entered human cells through the ACE 2 receptor, its first priority, similar to IAV, would be to enhance the HBP pathway to secure excessive glucose consumption and substrates for rapid replication. This abnormal HBP hyperactivity would lead to an excess of the OGT enzyme that would consequently trigger large amounts of IRF5 interferon. IRF5 and OGT will then coordinate efforts to exacerbate the IKK-NF-kB proinflamatory pathway triggering in the nucleus a massive inflammatory cytokine gene overexpression profile and a deleterious ER stress that ultimately result in hyperinflammation, a cytokine storm and multiorgan failure.

The detrimental consequences are not hard to imagine when this life-threatening hyperinflammatory HBP-GlcNAc-OGT–IRF5-interferon-SARS-CoV-2 pathway from the COVID-19 pandemic meets with CLGSPI found in common, complex highly prevalent pandemics such as obesity and diabetes, also found in not so old individuals with symptom-free adipose tissue dysfunction, insulin resistance, the metabolic syndrome and/or prediabetes (13, 47). These four groups of individuals present postprandial hyperglycemia which is one of the earliest abnormalities of glucose homeostasis associated with dysglycemic states (48). A recent publication highlighted the significance of angiotensin converting enzyme 2 (ACE2) and dipeptidyl peptidase-4 (DPP4), and their dual physiologic effects as transducers of metabolic signals regulating inflammation, cardiovascular physiology, and glucose homeostasis (49). This opens the door to consider glucose-lowering agents such as the DPP4 inhibitors as tools to intervene in the interaction of COVD-19, dysglycemic states and the HBP-GlcNAc-OGT–IRF5-interferon pathway, keeping in mind that DPP-4 inhibitors are not indicated in T1D individuals (50).

In summary, the HBP-GlcNAc-OGT–IRF5-interferon-SARS-CoV-2 pathway may have important implications regarding direct or indirect specific molecular targets for BCG activity (Figure 1). Importantly, dietary specific indications for low glycemic index diets (51, 52) instead of general dietary recommendations for COVID-19 infected individuals and/or at risk of infection may seem appropriate. This is supported by the hypothetical association between the large postprandial glucose peaks after daily meals, the HBP-GlcNAc-OGT–IRF5-interferon pathway and subjects with dysglycemia, adipose tissue dysfunction and low-grade subclinical inflammation at risk for severe COVID-19 infection.

## Author Contributions

RB and HL-M wrote the manuscript. IL-B edited and revised the manuscript. ER-A revised the final version. All authors have read and approved the final manuscript.

## Conflict of Interest

The authors declare that the research was conducted in the absence of any commercial or financial relationships that could be construed as a potential conflict of interest.
